# Role of spinal astrocytes through the perisynaptic astrocytic process in pathological pain

**DOI:** 10.1186/s13041-023-01069-z

**Published:** 2023-12-13

**Authors:** Hyoung-Gon Ko, Heejung Chun, Seunghyo Han, Bong-Kiun Kaang

**Affiliations:** 1 Department of Anatomy and Neurobiology, School of Dentistry, Kyungpook National University, 2177 Dalgubeol- daero, Daegu, 41940 South Korea; 2 Brain Science and Engineering Institute, Kyungpook National University, Daegu, South Korea; 3 College of Pharmacy, Yonsei-SL Bigen Institute (YSLI), Yonsei University, Incheon, South Korea; 4 Center for Cognition and Sociality, Life Science Institute, Institute for Basic Science (IBS), Daejeon, 34141 South Korea

**Keywords:** Astrocyte, Pathological pain, Spinal cord

## Abstract

Pathological pain is caused by abnormal activity in the neural circuit that transmits nociceptive stimuli. Beyond homeostatic functions, astrocytes actively participate in regulating synaptic transmission as members of tripartite synapses. The perisynaptic astrocytic process (PAP) is the key structure that allows astrocytes to play these roles and not only physically supports synapse formation through cell adhesion molecules (CAMs) but also regulates the efficiency of chemical signaling. Accumulating evidence has revealed that spinal astrocytes are involved in pathological pain by modulating the efficacy of neurotransmitters such as glutamate and GABA through transporters located in the PAP and by directly regulating synaptic transmission through various gliotransmitters. Although various CAMs contribute to pathological pain, insufficient evidence is available as to whether astrocytic CAMs also have this role. Therefore, more in-depth research is needed on how pathological pain is induced and maintained by astrocytes, especially in the PAP surrounding the synapse, and this will subsequently increase our understanding and treatment of pathological pain.

## Introduction

Pain is critical in warning of potential external hazards that threaten the survival of living organisms. However, repeated and uncontrollable pain that deviates from these positive functions impairs the quality of life. Changes in neuronal circuits transmitting sensory stimuli from the periphery to the central nervous system can occur in pathological pain states such as neuropathic and inflammatory pain [1]. Innocuous stimuli can induce nociception (allodynia), and weak nociceptive stimuli can be amplified (hyperalgesia) in these pathological conditions [2, 3]. Central sensitization is recognized as the underlying mechanism of hyperalgesia in the spinal cord [4]. Continuous nociceptive stimuli strengthen synaptic transmission in spinal neuronal circuits conveying corresponding sensory stimuli and thereby amplify nociception. The regulation of synaptic transmission is basically modulated in pre- and postsynaptic neurons. However, growing evidence suggests that astrocytes also actively participate in this process [5, 6].

Astrocytes in the central nervous system form a blood–brain barrier to control the entry and exit of molecules such as water and potassium ions from neurons. Astrocytes also remove excessive glutamate from the synaptic cleft to prevent cytotoxicity, and these functions help maintain homeostasis [5]. Beyond these pivotal functions, astrocytes can communicate with neurons and other astrocytes in several ways. Astrocytes release various gliotransmitters to modulate neuronal properties and express receptors to receive signals from pre- and postsynaptic neurons, and form unique structures, especially in the synaptic region, that effectively regulate synaptic transmission [7]. The perisynaptic astrocytic process (PAP) is the extension of astrocytic terminal processes toward synapses, where astrocytes wrap around the synaptic cleft [8–10]. This process is essential for modulating neurotransmitter signaling and synaptic plasticity since this regulates the extracellular space surrounding synapses. A key function of the PAP is to regulate neurotransmitter signaling [7], and astrocytes can uptake and recycle neurotransmitters, such as glutamate and GABA, which are involved in synaptic transmission. By controlling the availability of neurotransmitters in the synaptic cleft, astrocytes can modulate neurotransmitter signaling and, thereby influence synaptic plasticity. Furthermore, astrocytes express many types of cell adhesion molecules (CAMs) that interact directly with their neuronal binding partners located in pre- and postsynaptic regions [11, 12]. These glial transmembrane molecules physically anchor the pre- and postsynaptic membranes and support the integrity of the synaptic microdomain. Many studies have shown that glial CAMs also regulate synaptogenesis, dendritic arborization, and spine morphology [11–13].

As astrocytes are part of the tripartite synapse, their dysfunction is involved in many pathological states [14]. Specifically, pharmacological approaches such as the inhibition of glial metabolism using fluorocitrate suggest that spinal astrocytes are involved in pathological pain [15, 16]. Recently, optogenetic and chemogenetic tools demonstrated the requirement of spinal astrocytes in pathological pain [17, 18]. In this review, we briefly discuss how spinal astrocyte regulates synaptic transmission of nociceptive information. We then discuss in more detail the changes that occur in the PAP and how astrocytes regulate synaptic transmission of nociceptive stimuli through the PAPs in pathological pain conditions.

### Glial signaling molecules and gliotransmitters affecting pathological pain

Astrocytes in the spinal cord can respond to peripheral injury by releasing signaling molecules or peptides that affect pre- and postsynaptic terminals (Table 1). Expression of TNFα is enhanced in the spinal cord, including that from astrocytes after spinal nerve injury, and pretreatment with the TNFα synthesis inhibitor thalidomide prevents neuropathic pain development [19]. Release of glial TNFα strengthens excitatory synaptic transmission by increasing the insertion of AMPA receptors in the postsynaptic membrane [20]. In addition to postsynaptic mechanisms, TNFα can increase the release of presynaptic glutamates without affecting GABA release [21]. Although, the expression of TNFα in the hippocampus did not induce the insertion of the GABA receptor, this did induce endocytosis [22].

IL-1β is a recognized mediator of pathological pain released by spinal microglia; however, astrocytes also release IL-1β in pathological pain states [23]. Direct activation with optogenetic tools can induce IL-1β expression in spinal astrocytes [17]. Intrathecal injection of the IL-1β induces heat-mediated hyperalgesia and this effect of IL-1β is blocked when fluorocitrate, a glial metabolic suppressor, is administrated together [24]. Bath application of IL-1β phosphorylates NMDAR, thereby increasing Ca^2+^ entry through phosphorylated NMDAR, and finally induces C-fiber-mediated long-term potentiation (LTP) in spinal cord slices [24, 25]. Moreover, IL-1β can contribute to inducing pathological pain in the presynaptic mechanism, which enhances presynaptic glutamate release [21].

The next important glial factor for pathological pain is thrombospondin (TSP). After the initial discovery of its pivotal role in synaptogenesis, the expression of TSP was shown to be induced in the spinal cord by peripheral nerve injury [26, 27]. In pathological pain states, TSP is released from activated astrocytes to induce excitatory synaptogenesis and increase excitatory synaptic transmission in the spinal cord [28]. Knockdown of spinal TSP-4 expression attenuates mechanical allodynia and thermal hyperalgesia induced by spinal nerve ligation [27]. Collectively, peripheral nerve injury activates spinal astrocytes, which then express and release TSP-4 to induce excitatory spine formation, causing central sensitization and subsequent pathological pain.

Among the glial factors, the role of ATP in pathological pain is a more complex because of the diversity of its receptors [6]. Primarily, spinal astrocytes, together with microglia and neurons, can increase the exocytosis of ATP in response to peripheral injury [17, 29]. ATP that is released into the synaptic cleft can act as gliotransmitter for P2X (ligand-gated nonselective cation channel) and P2Y (ligand-gated G protein coupled receptor) classes of purinergic receptors located in pre- and postsynaptic regions. Synaptic ATP can be converted into adenosine, which also act as a ligand for P1 purinergic receptors (ligand-gated G protein coupled receptor) inducing pre- and postsynaptic changes [6]. In addition, adenosine can be directly released from astrocytes in the spinal cord without conversion from ATP [18]. Intrathecal injection of an antagonist or antisense oligonucleotide for the P2X receptor reduced neuropathic and inflammatory pain [30, 31]. Inhibition of the P2X receptor reduced neuronal excitability because this receptor is a ligand-gated cation channel [32]. P2Y receptors are mainly expressed in small-sized DRG neurons coexpressing TRPV1, especially the P2Y2 subtype, and positively regulate the function of the TRPV1 (+) nociceptor [33]. The P2Y1 receptor subtype also increases nociceptor excitability in DRGs, although this is based on different mechanisms from that of the P2Y2 receptor subtype. However, the P1 receptor (also known as an adenosine receptor, AR) can reduce synaptic transmission in the spinal cord, in contrast to the P2 receptor [34]. Recent studies showed that stimuli activating Aβ fibers, such as rubbing the damaged area, promote secretion from spinal astrocyte secretion of ATP that is quickly converted to adenosine, weakening the synaptic transmission of pain-related spinal neural circuits and reducing pain through P1 receptors [18].

D-serine is another important gliotransmitter related to chronic pain. It is synthesized by serine racemase and released from astrocytes, where it binds to the glycine binding site of the NMDA receptor as a coagonist [35]. Several reports have shown that the released D-serine enhances nociceptive transmission and mechanical allodynia through NMDA potentiation and neuronal NOS modulation in a neuropathic pain model of constriction injury [36–38]. Depletion of released D-serine by applying the D-serine-degrading enzyme, D-amino-acid oxidase, or the serine racemase inhibitor, L-serine O-sulfate, significantly alleviated mechanical allodynia in the chronic pain model [37, 39]. Interestingly, these effects were significantly reversed by exogenous D-serine applications [37]. Additionally, the alleviation of mechanical allodynia through fluorocitrate application, a glial metabolic suppressor, was reversed by exogenous D-serine [39, 40]. These studies imply that astrocyte-derived D-serine release controls pain-related synaptic transmission and chronic pain behavior.

Finally, intracellular Ca^2+^ and its related signaling in astrocytes help maintain the excitatory and inhibitory balance of neuronal activation [41]. In addition to ER-derived cytoplasmic Ca^2+^ in astrocytes, there are spontaneous Ca^2+^ activities in the distal processes of astrocytes (the “spotty Ca^2+^ microdomain”), which regulate extracellular concentrations of glio/neurotransmitters [42]. Astrocytic TRPA1 activation was shown to release glutamate in a Ca^2+^-dependent manner and maintain the expression of GAT3, a GABA transporter in astrocytes to ensure the correct concentration of extracellular GABA and inhibition of synaptic transmission [42, 43]. Additionally, astrocytic Ca^2+^ modulates the dynamic structural outgrowth or retraction of PAP, affecting the spine structural stability [44]. Previously, TRPA1 was reported to contribute to hyperalgesia in the dorsal horn region of the spinal cord [45, 46]. The pronociceptive actions of TRPA1 in the formalin-induced model of acute and chronic pain were shown to be significantly reversed by spinal administration of a TRPA1 antagonist [47]. However, as current studies are performed in knockout mice or using TRPA1 inhibitors, the cellular localization of TRPA1, whether in sensory neurons or astroglia, that contributes to pain sensation remains unclear. Therefore, the astrocytic control of Ca^2+^ signaling in PAPs via TRPA1 in the development of chronic pain needs to be clarified through experiments with cell-type specific TRPA1 modulation.

### Stabilization of spines by direct physical contact with the PAP

Astrocytes are an important component of the tripartite synapse because of the PAP, which prompts the question of how astrocytes regulate synaptic transmission in this “microspace” through the PAP. Apart from molecular signaling, astrocytes are involved in regulating synaptic transmission through direct physical contacts between PAPs and pre- and postsynaptic regions. LTP is reported to enhance glial coverage for excitatory synapse in the hippocampus, and whisker stimulation can increase glial coverage of the dendritic spine in the barrel cortex [48, 49]. Conversely, enhancement of glial coverage of the PAP after neuronal activity directly contributes to stabilization of dendritic spines [44, 50]. Based on the similarity between the molecular mechanisms of LTP and central sensitization, the tight contact between pre- and postsynaptic regions with PAPs could stabilize newly matured dendritic spines and thereby contribute to central sensitization [4, 10]. Evidence indicates that spinal cord injury increases mature dendritic spines in the spinal cord [51–53]. Furthermore, although intermediate filaments were not located in PAP regions [8, 9], infraorbital nerve injury enhances GFAP(+) fine and terminal astrocytic processes in the trigeminal nucleus [54]. Accumulating evidence indicates that enhancement of glial coverage by PAPs in the spinal cord may be induced and consequently contribute to pathological pain via spine stabilization; however, direct evidence for this causal relationship is currently lacking.

**Fig. 1:**
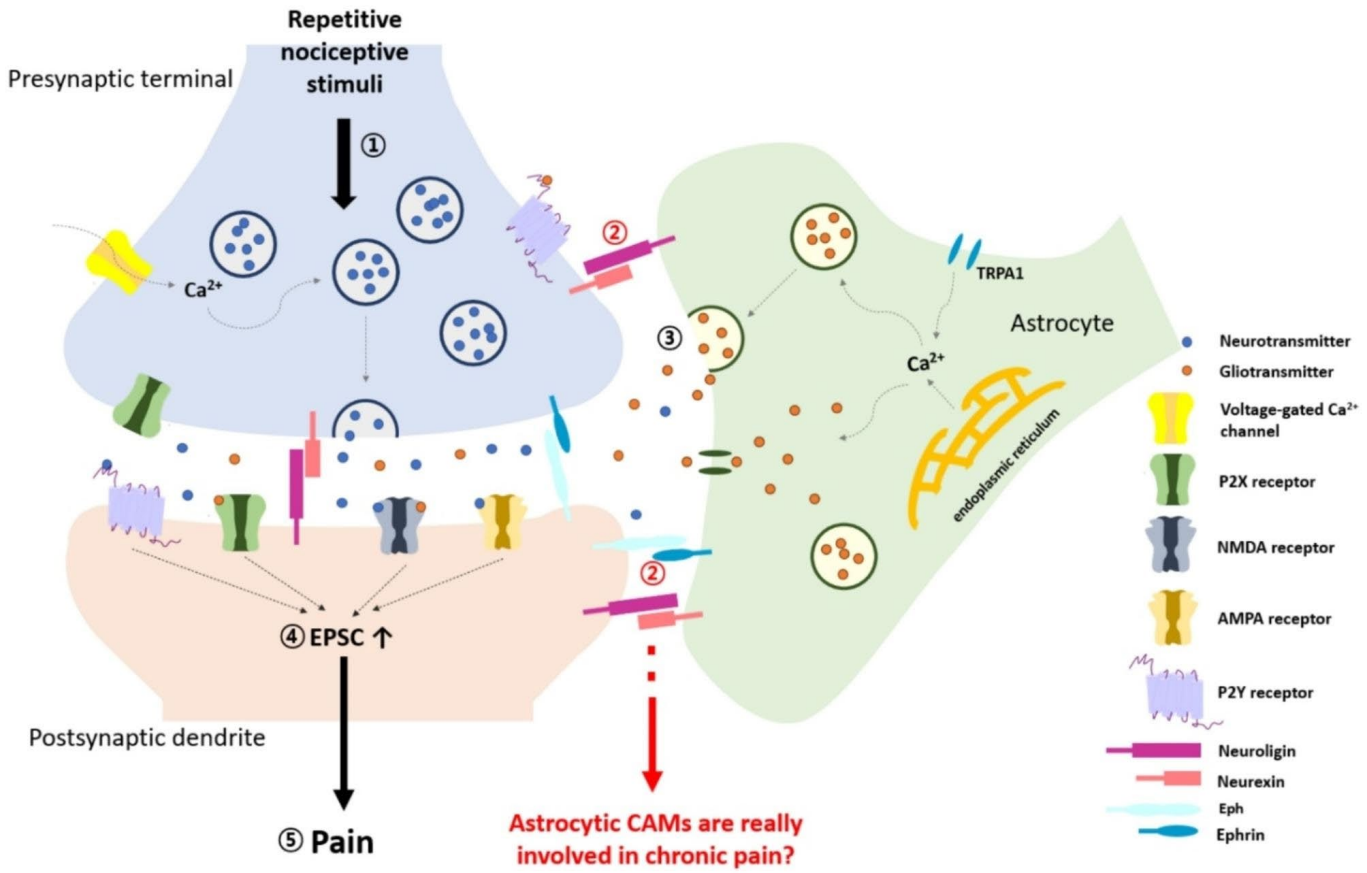
**Stabilization of spines by direct physical contact with PAP in pathological pain.** Schematic shows the direct and physical contacts between astrocytes and neuronal synapse. ① When repetitive nociceptive stimuli are transmitted in the spinal cord, the voltage-gated Ca^2+^ channel (VGCC) mediates Ca^2+^ influx in response to membrane depolarization and the intracellular Ca^2+^ triggers glutamate release from synaptic vesicles. ② Cell adhesion molecules (CAMs) such as neurexin-neuroligin or ephrin-Eph mediate direct physical contacts between PAPs and synaptic regions. ③ Gliotransmitters are released from PAPs and strengthen the synaptic transmission. Among them, the released ATP from astrocytes activates purinergic receptors such as P2X (ligand-gated nonselective cation channel) and P2Y (ligand-gated G protein coupled receptor) located in pre- and postsynaptic regions. ④ Increased glutamate in the synaptic cleft activates postsynaptic receptors such as AMPA and NMDA receptors, resulting in increased excitatory postsynaptic currents (EPSCs) and enhanced the synaptic transmission. ⑤ Finally, the body can recognize the pain in the chronic manner. However, it remains unclear whether astrocytic CAMs are really involved in chronic pain

The PAPs are directly anchored to the pre- or postsynaptic membrane through CAMs (Fig. 1). Thus, CAMs can regulate the formation of microdomains between the PAP and the pre- and postsynaptic membranes and subsequently in spine reorganization, synaptogenesis, and dendritic arborization [11, 12]. Most CAMs are expressed in both neurons and astrocytes, although some are specifically expressed in astrocytes. The neurexin–neuroligin interaction is well known in pre- and postsynaptic interactions; astrocytes also participate in the formation of tripartite synapses through these CAMs. Astrocytic neuroligin-2 affects the formation of excitatory synapse and the elimination of inhibitory synapse [55]. Moreover, astrocyte-specific deletion of neurexin-1 reduces the frequency of mEPSC, AMPAR-mediated EPSC, and LTP [56]. The ephrin–Eph interaction in glia-neuron communication is involved in regulating spine maturation. Glial ephrinA3 knockdown increases the mature spine and up-regulates the glial glutamate transporter [57]. In addition, the glial ephrinA3-neuronal Eph4 interaction is involved in LTP through regulation of the glial glutamate transporter to inhibit excessive glutamate toxicity [58].

These functions of CAMs ultimately produce regulation of synaptic transmission, and astrocytic CAMs can therefore also affect pathological pain. However, few studies have examined whether astrocytic CAMs are truly involved in chronic pain. Although it seems relatively clear that CAMs are necessary for the induction of pathological pain, most studies linking CAMs to pathological pain did not target astrocyte-specific CAMs [59–63]. Therefore, further research is needed to determine whether regulating the expression or function of CAMs specifically in astrocytes can affect pathological pain and, if so, how astrocytic CAMs modulate pathological pain.

### The PAP as a physical barrier maintaining glutamate and K^+^ in the synaptic cleft

Another way that glial coverage of PAPs can contribute to central sensitization is to increase the excitability of postsynaptic neurons by forming a physical barrier that retains glutamate without this leaking out of the synaptic cleft. After action is complete, however, glutamates released from the presynaptic terminal must be quickly removed to prevent excitotoxicity. The PAPs are capable of playing this protective role in the basal state. Glutamate aspartate transporter (GLAST) and glutamate transporter-1 (GLT-1) are glutamate transporters specifically expressed in astrocytes and not in neurons that help to clear excessive levels of glutamate remaining in the synaptic cleft [64]. After being transported into astrocytes, glutamates are converted into glutamine by glutamine synthetase (GS), which is predominantly expressed in astrocytes; these glutamines are then released and reused by the glutamatergic terminal. In pathological pain conditions such as CCI, the expression of GLAST and GLT-1 is downregulated in the spinal cord [65]. Inhibition of glutamine presynaptic reuptake by glutamatergic neurons hinders central sensitization induced by mustard oil [66]. Intrathecal injection of methionine sulfoximine, a GS inhibitor, also produces results similar to those of glutamine reuptake inhibitors [67]. Another interesting point is that the expression of the GABA transporter in astrocytes is increased in inflammatory pain [68]. How astrocytes strike a balance between increasing excitability by extending glutamate residence time in the synaptic cleft and preventing excitotoxicity by excessive glutamates, however, is still unknown.

In addition to the regulation of extracellular glutamate levels, astrocytes maintain the homeostatic concentration of K^+^ in the extracellular space by sensing neuronal activity in a process known as K^+^ buffering [69]. Pain-inducing sensory stimulation triggers action potentials in neurons of the pain pathway, causing the outflow of K^+^ ions into the extracellular space. Correct elimination of extracellular K^+^ ions is critical for restoring the membrane potential of neurons to enable the next action potential and precise control of sensory information processing. In the PAP, astrocytes express a specific K^+^ channel, Kir4.1, which is an inward-rectifying background K^+^ channel in astrocytes and is also detected in spinal astrocytes [70]. Single-cell RNA sequencing analysis was used to show that the expression level of Kir4.1 was significantly decreased in spinal astrocytes in the CCI mouse model [71]; selective genetic inhibition of the astrocytic Kir4.1 channel in the spinal cord induced hyperalgesia, whereas the overexpression of this channel reversed the effect. Furthermore, these astrocytic modulations of Kir4.1 significantly affected neuronal firing patterns in the dorsal spinal cord [71], implying that astrocytic regulation of K^+^ ions is an important factor in pain information processing.

**Fig. 2:**
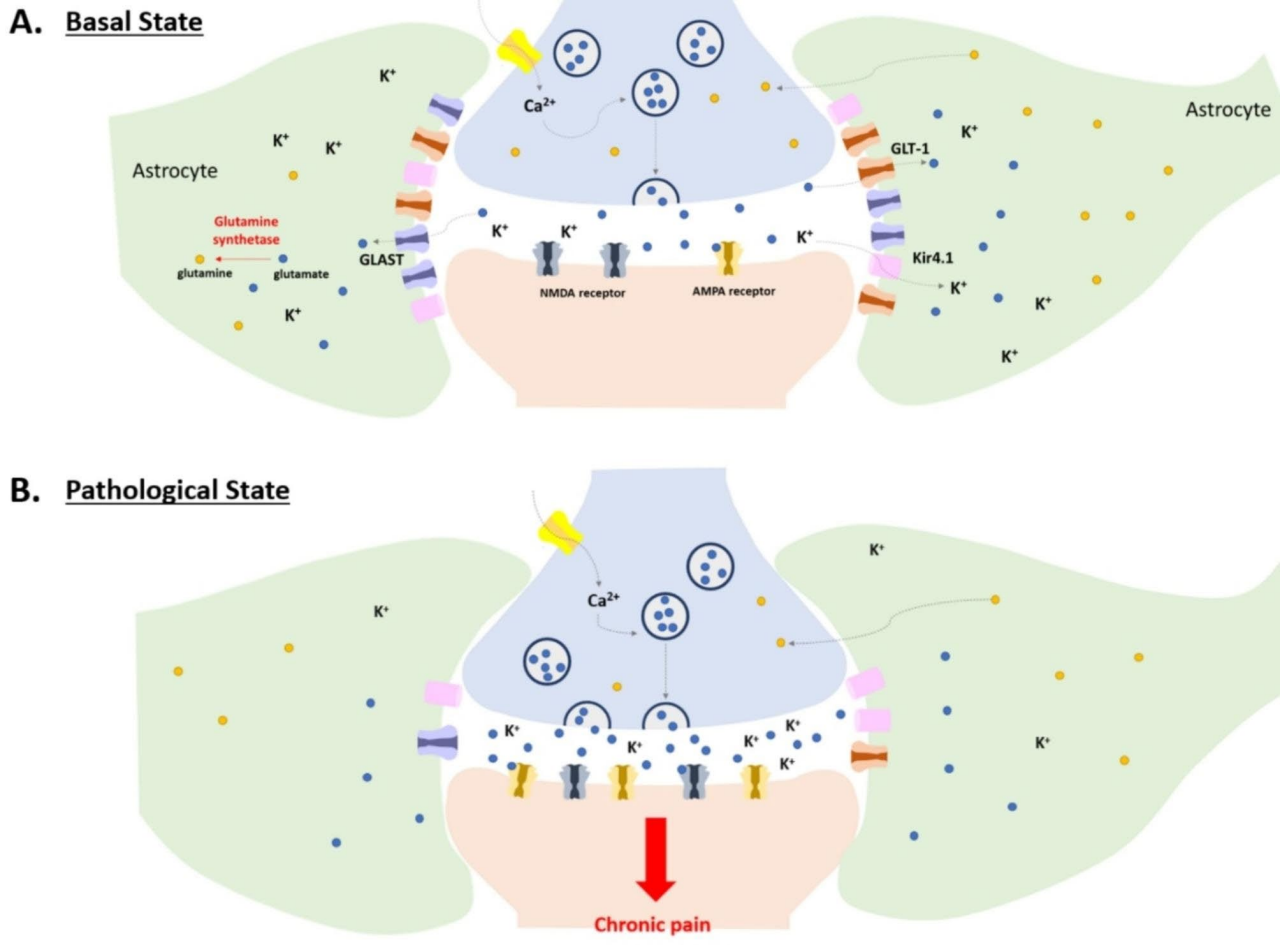
**PAPs as physical barrier keeping glutamates and K**^**+**^ **in synaptic cleft at pathological state of pain. A**, In the basal state, glutamate aspartate transporter (GLAST) and glutamate transporter-1 (GLT-1), which are specifically expressed in astrocytes, clear the excessive glutamates remained in the synaptic cleft. After being transported into astrocytes, glutamates are converted into glutamines by glutamine synthetase (GS). In addition, astrocytes maintain the homeostasis of K^+^ concentration in extracellular space through a specific K^+^ channel, Kir4.1. The glial coverage and the regulated mechanism make synaptic transmission accurate. **B**, In the pathological pain states, the expression of GLAST, GLT-1, and Kir4.1 is decreased, while the PAP’s encapsulation remains stable. These changes lead to the accumulation of glutamates and K^+^ ions in the synaptic cleft. Consequently, postsynaptic membranes become more excitable and transfer the pain signal abnormally

Collectively, reinforced encapsulation of PAPs in pathological pain states can dynamically regulate the excitation/inhibition balance through transporters expressed on PAPs beyond the role of a simple physical barrier (Fig. 2). In addition to regulating the efficacy of synaptic transmission, the glial coverage could strengthen the accuracy of synaptic transmission. As mentioned above, PAPs construct a physical barrier surrounding the synaptic clefts, and without this barrier, neurotransmitters can spill over into adjacent synaptic regions, thereby affecting the uncontrolled activities of unspecific neurons that are not related to specific neurons transmitting the corresponding nociceptive information [7, 72]. Therefore, nociceptive signals can be amplified by an enhanced signal-to-noise level.

## Conclusions and perspectives

In this review, we summarized the roles of and changes in spinal astrocytes, focusing on PAPs in pathological pain conditions. Growing evidence indicates that tight encapsulation of the synaptic region is required for pathological pain; however, direct evidence is lacking because of the absence of a technique that can assess the specific regulation of PAPs. In addition, physiological or pathophysiological stimulation does not always enhance synaptic transmission through tight glial coverage of PAPs. For instance, in cases of lactation, sucking stimuli decrease glial coverage in the supraoptic nuclei, and thereby increase the firing of POMC neurons in the arcuate nucleus [73]. Moreover, glial coverage is not only restricted to the perisynaptic region and can also include functions and changes in the neuronal soma, responding to pathological states [73, 74]. Thus, care needs to be taken when interpreting the change in glial coverage. Future studies involving loss-of- and gain-of-function experiments based on more specific tools that can assess PAP regulation will help contribute to our understanding of the role of PAPs in pathological pain.

**Table 1:** **Glial signaling molecules and gliotransmitters affecting pathological pain.** The table summarizes gliotransmitters released from spinal astrocytes that have important effects on pathological pain. The gliotransmitter-related mechanisms that strengthen or weaken pathological pain are described

	(+): strengthen		(-): weaken	
**Gliotransmitter**	**Proposed Functions**		**Roles in pathological pain**	**Reference**
**TNFα**	Excitatory transmission (+), Insertion of AMPA receptor (+), Presynaptic glutamate release (+), GABA receptor endocytosis (+)		Inhibition: development of neuropathic pain	[19–22]
**IL-1β**	Phosphorylation of NMDA receptor (+), Ca^2+^ entry (through NMDAR) (+), C-fiber-mediated long-term potentiation (+), Presynaptic glutamate release (+)		Inhibition: allodynia, Enhancement: hyperalgesia	[17, 21, 23–25]
**thrombospondin (TSP)**	Excitatory synaptogenesis (+), Excitatory synaptic transmission (+)		Inhibition: mechanical allodynia and thermal hyperalgesia	[26–28]
**ATP**	Excitability of nociceptor in DRG (+)		Inhibition: analgesic effect	[6], [17], [18], [30–33]
**Adenosine**	Synaptic transmission (-)		Enhancement: analgesic effect	[6], [18], [34]
**D-serine**	Potentiation of NMDA receptor (+), Increase of neuronal NOS (nNOS) activity (+), Nociceptive transmission (+), Chronic pain behavior (+)		Inhibition: mechanical allodynia	[35–40]
**TRPA1/Ca** ^**2+**^ **-mediated gliotransmitters**	Maintain the excitatory and inhibitory balance of neuronal activation (+) Astrocytic glutamate release (+), Maintain expression of GAT3(GABA transporter in astrocyte) (+), Modulate the astrocytic structural outgrowth or retraction of PAP.		Inhibition-TRPA1: acute and chronic pain (-)	[41–47]

## Data Availability

Not applicable. No data was generated during the current study.
